# Müllerian Anomalies Prevalence Diagnosed by Hysteroscopy and Laparoscopy in Mexican Infertile Women: Results from a Cohort Study

**DOI:** 10.3390/diagnostics9040149

**Published:** 2019-10-17

**Authors:** Enrique Reyes-Muñoz, Salvatore Giovanni Vitale, Deisi Alvarado-Rosales, Esther Iyune-Cojab, Amerigo Vitagliano, Franziska Michaela Lohmeyer, Yenara Patricia Guevara-Gómez, Alma Villarreal-Barranca, José Romo-Yañez, Araceli Montoya-Estrada, Fela Vanesa Morales-Hernández, Patricia Aguayo-González

**Affiliations:** 1 Department of Gynecological and Perinatal Endocrinology, Instituto Nacional de Perinatología, Mexico City 11000, Mexico; dr.enriquereyes@gmail.com (E.R.-M.); jryz@yahoo.com (J.R.-Y.); ara_mones@hotmail.com (A.M.-E.); 2 Unit of Gynecology and Obstetrics, Department of General Surgery and Medical Surgical Specialties, University of Catania, 95123 Catania, Italy; 3 Division of Human Reproduction, Instituto Nacional de Perinatología, Mexico City 11000, Mexico; dey0709@hotmail.com (D.A.-R.); estheriyu86@gmail.com (E.I.-C.); yenaraguevara@hotmail.com (Y.P.G.-G.); vanesamoh@gmail.com (F.V.M.-H.); pagonzalez2108@yahoo.com.mx (P.A.-G.); 4 Unit of Gynecology and Obstetrics, Department of Women and Children’s Health, University of Padua, 35122 Padua, Italy; amerigo.vitagliano@gmail.com; 5 Scientific Directorate, Fondazione Policlinico Universitario A. Gemelli IRCCS, 00168 Rome, Italy; franziskamichaela.lohmeyer@policlinicogemelli.it; 6 Faculty of Medicine Universidad Veracruzana, Veracruz 91090, Mexico; alma.vvb@gmail.com

**Keywords:** Müllerian anomalies, congenital uterine anomalies, infertility, prevalence

## Abstract

Background: To evaluate the prevalence of Müllerian anomalies (MAs) in a cohort of infertile Mexican women candidates for infertility treatments (intrauterine insemination or IVF (In vitro fertilization) cycles). Methods: We performed a retrospective observational study on a cohort of consecutive women, who underwent hysteroscopy and laparoscopy as part of the basic infertility workup from 2002 to 2014, at our center. Our aim was to calculate the prevalence of MAs and each subtype. Results: A total of 4005 women were included in the study. The MA prevalence was 4.4% (95% CI; 3.8–5.1; *n* = 177). Among women with MAs, the prevalence of different MA types was: septate uterus 54.2% (*n* = 96), arcuate uterus 15.8% (*n* = 28), bicornuate uterus 10.7% (*n* = 19), unicornuate uterus 8.5% (*n* = 15), didelphys uterus 6.2% (*n* = 11) and hypoplasia/agenesis 3.4% (*n* = 6), unclassifiable 1.1% (*n* = 2). Women with MAs who achieved pregnancy were: 33.3% (*n* = 59). The MA associated with the highest pregnancy rate was septate uterus after hysteroscopic correction, at 38.5% (37/96). Conclusions: The prevalence of MAs among infertile Mexican women can be considered as low, but not negligible. The septate uterus is the most common MA in women with infertility.

## 1. Introduction

Normal female reproductive tract development involves specific and complex processes characterized by differentiation, migration, fusion and canalization of the Müllerian duct [1,2]. Müllerian anomalies (MAs) include a group of congenital anomalies resulting from alterations in the embryonic development of the Müllerian ducts, potentially affecting the morphology of the uterus, fallopian tubes, cervix and vagina. MAs can sometimes be associated with ovarian, urinary tract, skeletal, or other organ anomalies [3,4,5,6,7].

The first attempt at systematically classifying MAs was made in 1907; later, many other classifications of MAs were proposed [8]. In 1979, Buttram and Gibbons developed a classification system based on female genital tract development [9]. The American Society of Reproductive Medicine (ASRM) reviewed and modified this classification in 1988, categorizing seven different MA types [10]. ASMR classification of MAs is well established, due to its overall simplicity, focusing on uterine anomalies in relation with fertility [3,4]. However, in 2004, another classification system, based on the embryologic origin of the different genital tract organs, was proposed [11,12].

MAs are associated with higher obstetric complication risks, including recurrent pregnancy loss (RPL) and preterm delivery, as well as higher perinatal morbidity and mortality [13,14,15]. Some authors speculate on the possible role of MAs in the pathophysiology of female infertility, but a causal relationship has not yet been demonstrated [16,17]. Sometimes, MAs may remain asymptomatic, and are only diagnosed accidentally in multiparous women [18]. 

Given the rarity of MAs, as well as the lack of a universally accepted classification system, and the different diagnostic strategies in common use (with variable diagnostic accuracy) [19,20,21,22], it is currently difficult to estimate the real prevalence of MAs. In this regard, a recent systematic review reported a prevalence of 5.5% in the general population, while its prevalence among infertile women was slightly higher (about 8.0%). The limitations of this review, by Chan et al. [19], were related to the substantial heterogeneity of the patients, as well as to the applied diagnostic tests and the classification systems used.

Based on this, we aimed to determine the prevalence of MAs and of MA subtypes (classified following the ASRM classification) in a consecutive cohort of Mexican infertile women who underwent diagnostic hysteroscopy and laparoscopy as a basic infertility workup. Secondarily, we evaluated the reproductive outcomes of these patients during the study period.

## 2. Materials and Methods 

### 2.1. Study Design and Ethical Approval

This was a retrospective observational study performed at Instituto Nacional de Perinatología, Isidro Espinosa de los Reyes, in Mexico City. We enrolled all women who underwent a hysteroscopy and laparoscopy during their infertility evaluation at our infertility clinic between January 2002 and December 2014. Data were obtained from clinical records and incomplete files were excluded. Our Internal Review Board approved the study (registry number: 2019-1-43). 

### 2.2. Study Objectives

The primary outcome was to assess the prevalence of MAs and MA subtypes in the study cohort. The secondary outcome was to evaluate the percentage of women with MAs who achieved pregnancy.

### 2.3. Patients and Procedures

All the enrolled patients were diagnosed with infertility, defined as the incapability to achieve a pregnancy after ≥12 months of sexual intercourse without contraceptives [23]. Inclusion criteria were infertile woman candidate for intrauterine insemination or IVF/ICSI (Intracytoplasmatic Sperm Injection), having submitted to hysteroscopy and laparoscopy before assisted reproductive techniques (ARTs). Exclusion criteria were incomplete medical records, and suspected MAs by means of sonohysterography (SHG) only, without hysteroscopy/laparoscopy.

All women underwent hormonal tests (LH (Luteinizing hormone), FSH (Follicle-stimulating hormone), estradiol, prolactin), thyroid function test, fasting glucose, vaginal culture, chlamydia trachomatis detection by PCR, ureaplasma urealyticum and mycoplasma hominis culture, endovaginal ultrasound, followed by a hysterosalpingography (HSG). In select cases, an SHG was performed. Two authors collected data from medical records about general patient features and information on patients’ follow-up. We systematically recorded the following variables during the first clinical visit: age, number of previous gestations, miscarriages, type of infertility, weight, height and body mass index (BMI) calculated using the equation: weight (kg)/height (m^2^). Primary infertility was considered in the absence of a previous pregnancy, and secondary infertility in cases with a confirmed previous pregnancy. Pregnancy was defined as a positive pregnancy test, plus the identification of a gestational sac at the ultrasound examination and/or the presence of an embryo with heartbeat. 

Immediately after diagnostic workup, hysteroscopic correction was performed with a laparoscopy operative guide, using hysteroscopic scissors, monopolar energy and/or bipolar energy (VersaPoint system. Gynecare; Ethicon Inc., NJ, USA) [5,10,24]. The first ART attempt was performed about 6–12 months after hysteroscopic correction. A second attempt was performed 6–24 months after the first attempt. Many women spontaneously conceived during the waiting time between the first and the second ART attempt. All the women were followed up until December 2014. 

### 2.4. Definition and Diagnosis of Müllerian Anomalies

MAs were defined as congenital anomalies of the Müllerian duct, affecting uterus, tubes, cervix and/or vaginal morphology [1]. Reference standard for diagnosis was hysteroscopy and laparoscopy, performed in the operating room under general anesthesia. MA types were differentiated according to ASRM classification [10]. 

### 2.5. Sample Size

The sample size was calculated by considering an estimated prevalence of MA of 5%. A sample of 3112 patients was necessary to obtain an estimate precision of 1% with a confidence level of 1 − α = 99%.

### 2.6. Statistical Analysis

Statistical analysis was performed using the Statistical Package for Social Sciences Software (SPSS V.15, Chicago, IL, USA). Continuous variables were expressed as mean ± standard deviation, and categorical variables as absolute frequencies and percentages, according to data distribution. Prevalence was calculated with a 95% confidence interval.

## 3. Results

A total of 4005 infertile women were submitted for hysteroscopy and laparoscopy between January 2002 and December 2014. A total of 177 women (4.4%; CI 95%; 3.8–5.1) were diagnosed with MA. The majority of patients with MA had primary infertility (*n* = 122, 68.9%). Table 1 shows the general characteristics of women with a diagnosis of MA.

As showed in Table 2, septate uterus was the most prevalent type of MA (*n* = 96, 54.2%). The other MA types diagnosed were arcuate uterus (*n* = 28, 15.8%), bicornuate uterus (*n* = 19, 10.7%), unicornuate uterus (*n* = 15, 8.5%), didelphys uterus (*n* = 11, 6.2%), uterine hypoplasia/agenesis (*n* = 6, 3.4%). Among the women with uterine hypoplasia/agenesis, one was diagnosed with Mayer-Rokitansky-Küster-Hauser syndrome; two cases (1.1%) were unclassifiable according to the ASRM system. Five (2.8%) renal anomaly cases were associated with MA.

Hysteroscopic corrections were performed in 102 women (60.4%), while 75 (39.6%) women remained without hysteroscopic correction. Hysteroscopic correction was performed using hysteroscopic scissors (*n* = 64, 62.7%), monopolar energy (*n* = 8, 7.8%) and bipolar energy (*n* = 30, 29.4%). The most frequent hysteroscopic procedure was metroplasty (*n* = 96, 94.1%).

Of 177 women diagnosed with MA, 33.3% (*n* = 59) achieved pregnancy (Table 3).

The MA types associated with higher pregnancy rates were septate uterus (38.5%), unicornuate (33.3%) and arcuate (32.1%) uterus. The majority of pregnancies were achieved spontaneously (Table 4).

## 4. Discussion

The aim of our study was to evaluate the prevalence of MAs in a cohort of infertile Mexican women candidates for infertility treatments (intrauterine insemination or IVF cycles). We found that the MA prevalence in our cohort was 4.4%. In the present study, MA diagnosis was achieved by means of hysteroscopy and laparoscopy, currently considered the gold standard for the diagnosis of MAs [15]. The high accuracy of the diagnostic methods employed could potentially explain the lower prevalence of MA found in our study, compared to other studies [20,25,26]. Similarly, Raga et al. evaluated the morphology of the uterus by hysterosalpingography (HSG) and laparoscopy/laparotomy. The overall frequency of uterine malformations in their study population was 4.0%. Incidences of MA were higher in infertile women (6.3%), compared to fertile (3.8%) and sterile (2.4%) ones [26]. However, Acien et al. reported a considerably higher prevalence of MA (16%) in the Spanish population [25]. Nevertheless, MAs were diagnosed mainly by transvaginal ultrasound and HSG, and only 28% of diagnoses were confirmed by laparoscopy/laparotomy, potentially increasing the rate of false positive diagnoses.

In a systematic review by Saravelos et al., the sensitivity of different diagnostic methods was considered. Studies were grouped into three classes: Class Ia hysteroscopy and laparoscopy, Ib only hysteroscopy, II; HSG or 2D ultrasound and III; magnetic resonance imaging (MRI) and physical examination during pregnancy or delivery. The authors reported a prevalence of 7.3% (95% CI 6.7–7.9) in women with infertility of class Ia and Ib; however, the prevalence of MA in class II was 10.8% [20]. The most frequent MAs in women with infertility were septate (54.2%), arcuate (15.8%) and bicornuate (10.8%) uterus, similar to other studies. Raga et al. found that the most frequent MA were septate (33.6%), arcuate (32.8%) and bicornuate (20.3%) uterus [26]; Saravelos et al. reported the same MA in a proportion of 4:2:1, respectively [20].

In line with our results, the review by Chan et al. reported canalization defects (including septate and subseptate uterus) as the most common MA, with a prevalence in the general population of 2.3% (95% CI 1.8–2.9). However, no significantly higher MA rates in infertile women (3.0%; 95% CI 1.3–6.7; *p* = 0.422) were reported. Notably, the prevalence of MAs was higher in women with an RPL history (5.3%; 95% CI 1.7–16.8; *p* = 0.021) and in those with RPL and infertility (15.4%; 95% CI 12.5–19.0). The second and third most common types of MA among infertile women were arcuate uterus (1.8%; 95% CI 0.8–4.1) and bicornuate uterus (1.1%; 95% CI 0.6–2.0), respectively [19]. These results are similar to those of our study.

Another interesting aspect of our study concerns renal anomalies. A study conducted in 2007 by Oppelt et al. found other anomalies associated with about 36% of MA cases, and the most frequent association was with renal anomalies [27]. We found a prevalence of 2.8% of renal anomalies associated with MAs.

The association between MAs and RPL is well described [27,28]; however, its association with infertility is controversial [29,30]. Chan et al. reported no difference in pregnancy rates when comparing women with MAs and women with normal uteri (RR: 0.87 95% CI 0.68–1.11; *p* = 0.25) [16]. The meta-analysis by Venetis et al. reported the effects of MAs on reproductive outcomes. The authors concluded that septate uterus was the only MA associated with a significant decrease in the natural conception rate (RR: 0.86; 95% CI; 0.77–0.96) [31]. In the absence of a control group, no direct inferences between MAs and infertility can be drawn. Nevertheless, the prevalence of septate uterus in our infertile cohort was considerably higher, compared to other data in the general population, potentially suggesting an association with infertility.

In the current study, 33.3% of women with MAs and infertility achieved pregnancy, with a higher percentage among women with septate uterus (38.5%). It should be mentioned that these women underwent a septum resection by hysteroscopy before performing any ART or achieving spontaneous pregnancy, while women with other MAs did not receive any surgical treatment.

In 2013, Valle et al. evaluated the effect of the septum resection in women with infertility and reported a pregnancy rate of 67.8% (95% CI 62.5–72.8), with a live birth rate of 53.5% (95% CI 43.4–57.1) [32]. Jayaprakasan et al. reported that pregnancy rates in women who underwent ART were similar in women with an arcuate uterus (54.5%), major uterine anomalies (70%) and normal uterus (43.4%) [33]. In the present study, the pregnancy rate was lower than in previous studies.

The strengths of our study are the large sample size, the use of laparoscopy and hysteroscopy in all patients and the homogeneity of the study population (i.e., only patients with a diagnosis of infertility). The main limitation of our study is its retrospective design within a single ethnic group.

## 5. Conclusions

Based on hysteroscopy and laparoscopy, we found a low but non-negligible prevalence of MAs among infertile patients. Septate uterus was the most frequent MA. As our findings refer to a Mexican group of infertile patients, they may not be generalizable to other ethnic groups.

## Figures and Tables

**Table 1:** Characteristics of Mexican women with Müllerian anomalies.

Characteristics	*n* = 177
Age (years)	28.9 ± 4.2
Weight (kg)	64.4 ± 9.9
Height (m)	1.55 ± 0.6
BMI (kg/m^2^)	26.7 ± 3.7
Primary infertility	122 (68.9%)
Secondary infertility	55 (31.1%)

MA: Müllerian Anomalies; BMI: body mass index. Data are expressed as mean ± SD or absolute frequency (%).

**Table 2:** Absolute frequency of Müllerian anomalies according to the ASRM classification.

Müllerian Anomalies	*n* = 177	Prevalence (%) in 4005 Women by Hysteroscopy & Laparoscopy
I: Hypoplasia/Agenesis	6 (3.4)	0.15
Ia: Vaginal	0 (0)	0
Ib: Cervix	0 (0)	0
Ic: Fundus	5 (2.8)	0.12
Id: Tubarian	0 (0)	0
Ie: Mixed	1 (0.6)	0.02
II: Unicornuate	15 (8.5)	0.37
IIa: Communicant	4 (2.3)	0.10
IIb: No communicant	3 (1.7)	0.07
IIc: Without cavity	3 (1.7)	0.07
IId: Without horn	5 (2.8)	0.12
III: Didelphys	11 (6.2)	0.27
IV: Bicornuate	19 (10.7)	0.47
IVa: Complete	7 (4.0)	0.17
IVb: Parcial	12 (6.8)	0.30
V: Septate	96 (54.2)	2.40
Va: Complete	17 (9.6)	0.42
Vb: Parcial	79 (44.6)	2.00
VI: Arcuate	28 (15.8)	0.70
VII: DES	0 (0)	0
No classifiable	2 (1.1)	0.05
Total	177 (100)	4.41

Data are expressed as frequencies (%). ASRM: American Society of Reproductive Medicine; DES: Diethylstilbestrol.

**Table 3:** Percentages of achieved pregnancy.

Müllerian Anomalies	Pregnancy	No Pregnancy
I: Hypoplasia/Agenesis *n* = 6	0 (0)	6 (100)
II: Unicornuate *n* = 15	5 (33.3)	10 (66.7)
III: Didelphys *n* = 11	3 (27.3)	8 (72.7)
IV: Bicornuate *n* = 19	5 (26.3)	14 (73.7)
V: Septate *n* = 96	37(38.5)	59 (61.5)
VI: Arcuate *n* = 28	9 (32.1)	19 (67.9)
VII: DES *n* = 0	0 (0)	0 (0)
Nonclassifiable *n* = 2	0 (0)	2 (100)
Total *n* = 177	59 (33.3)	118 (66.7)

Data are expressed as frequencies (%). DES: Diethylstilbestrol.

**Table 4:** Methods used to achieve pregnancy in 59 Mexican Müllerian anomaly women.

Method	Absolute Frequency and %
Spontaneous	32 (54.2)
Ovulation Induction	10 (17.0)
Intrauterine Insemination	8 (13.6)
IVF/ICSI	9 (15.2)

IVF: in vitro fertilization; ICSI: intracytoplasmic sperm injection.
